# Supplementary figures and images for: The unexpected effect of the compound microbial agent NP-M2 on microbial community dynamics in a nonylphenol-contaminated soil: the self-stability of soil ecosystem
Source: PeerJ. 2024 May 30;12:e17424. doi: 10.7717/peerj.17424 (PMC11144391; doi:10.7717/peerj.17424)

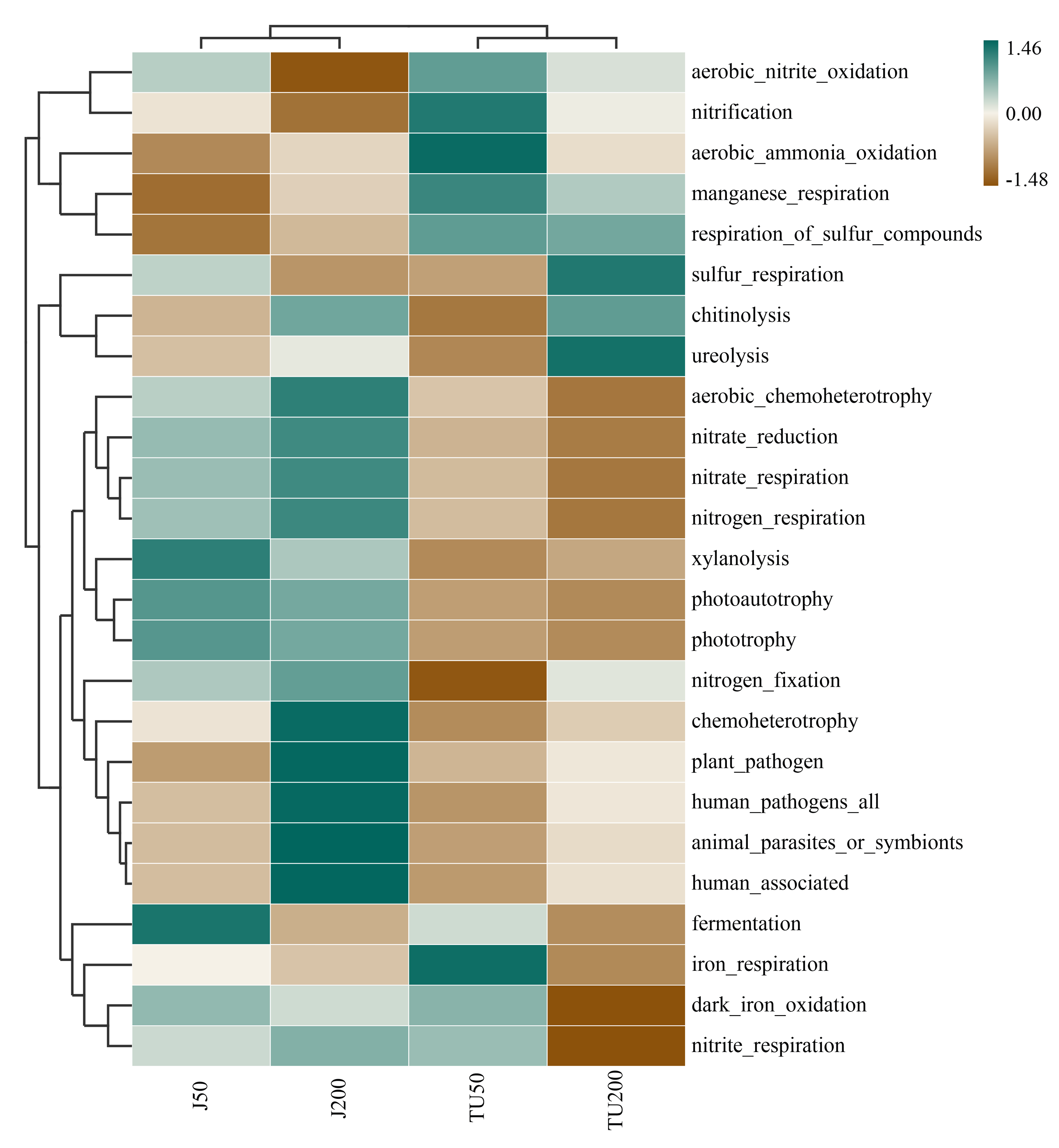

Supplement: Supplemental Information 1 [file peerj-12-17424-s001.png]
