# Supplementary material for: The unexpected effect of the compound microbial agent NP-M2 on microbial community dynamics in a nonylphenol-contaminated soil: the self-stability of soil ecosystem
Source: PeerJ. 2024 May 30;12:e17424. doi: 10.7717/peerj.17424 (PMC11144391; doi:10.7717/peerj.17424)
Supplement: Supplemental Information 2 — Residuals, the parts that cannot be explained by these factors; SumOfSqs, the total variance of each element in each grouping factor; F. Models, the statistical F-value; R2, the explanation degree of the differences between samples by the grouping factors; Pr (>F), the P-value of significance of the differences, and P < 0.05 represents the significant effect. [file peerj-12-17424-s002.docx]

**Table S1** **Permutational multivariate analysis of variance (PERMANOVA) analysis of the effects of NP concentration and microbial agent addition.**

| Items | SumOfSqs | F.Models | R^2^ | Pr (>F) |
| --- | --- | --- | --- | --- |
| NP Concentration | 0.370035 | 1.341254 | 0.092588 | 0.001 |
| NP-M2 addition | 0.315875 | 1.144941 | 0.079037 | 0.009 |
| NP Concentration*NP-M2 addition | 1.010098 | 1.240163 | 0.252742 | 0.001 |
| Residuals | 2.300547 | － | 0.575633 | － |
| Total | 3.996555 | － | 1 | － |

Residuals: the parts that cannot be explained by these factors; SumOfSqs: the total variance of each element in each grouping factor; F.Models: the statistical F-value; R^2^: the explanation degree of the differences between samples by the grouping factors; Pr (>F): the P-value of significance of the differences, and P<0.05 represents the significant effect.
